# Risk characterization of hospitalizations for mental illness and/or behavioral disorders with concurrent heat-related illness

**DOI:** 10.1371/journal.pone.0186509

**Published:** 2017-10-16

**Authors:** Michael T. Schmeltz, Janet L. Gamble

**Affiliations:** 1 ASPPH/EPA Environmental Health Fellowship Program at the United States Environmental Protection Agency, Office of Research and Development, National Center for Environmental Assessment, Washington, DC, United States of America; 2 United States Environmental Protection Agency, Office of Research and Development, National Center for Environmental Assessment, Washington, DC, United States of America; Columbia University, UNITED STATES

## Abstract

**Background:**

Many studies have found significant associations between high ambient temperatures and increases in heat-related morbidity and mortality. Several studies have demonstrated that increases in heat-related hospitalizations are elevated among individuals with diagnosed mental illnesses and/or behavioral disorders (MBD). However, there are a limited number of studies regarding risk factors associated with specific mental illnesses that contribute, at least in part, to heat-related illnesses (HRI) in the United States.

**Objective:**

To identify and characterize individual and environmental risk factors associated with MBD hospitalizations with a concurrent HRI diagnosis.

**Methods:**

This study uses hospitalization data from the Nationwide Inpatient Sample (2001–2010). Descriptive analyses of primary and secondary diagnoses of MBDs with an HRI were examined. Risk ratios (RR) were calculated from multivariable models to identify risk factors for hospitalizations among patients with mental illnesses and/or behavioral disorders and HRI.

**Results:**

Nondependent alcohol/drug abuse, dementia, and schizophrenia were among the disorders that were associated with increased frequency of HRI hospitalizations among MBD patients. Increased risk of MBD hospitalizations with HRI was observed for Males (RR, 3.06), African Americans (RR, 1.16), Native Americans (RR, 1.70), uninsured (RR, 1.92), and those 40 years and older, compared to MBD hospitalizations alone.

**Conclusions:**

Previous studies outside the U.S. have found that dementia and schizophrenia are significant risk factors for HRI hospitalizations. Our results suggest that hospitalizations among substance abusers may also be an important risk factor associated with heat morbidity. Improved understanding of these relative risks could help inform future public health strategies.

## Introduction

According to the most recent United States National Climate Assessment, mean temperatures are likely to increase by 2°F to 4°F in the U.S. over the next few decades with higher estimates in the range of 5°F to 10°F by the end of the century, given higher emission scenarios [1]. Elevated temperatures have the potential to increase occurrences of water-borne diseases, extreme heat events, and prolonged drought and wildfire conditions, all of which affect human health and livelihood [2,3]. Acute and chronic psychological impacts associated with extreme weather events may be attributed to stress, anxiety, depression, and other mental health problems, which can linger for months or even years, though these underlying conditions may also be exacerbated by extreme events [4,5]. The impact of extreme events can include physical injuries and mortality caused by heatwaves, floods, and vector-borne diseases, though these events also pose a serious risk to mental health and societal wellbeing [6,7]. Berry, et al (2010) describe a causal framework that explains how direct and indirect impacts from climate change will impact mental health by exposing people to psychological and physical trauma associated with extreme events as well as impacts to community well-being by damaging the economy and social fabric of communities [8]. Extreme heat events, in particular, are known to exacerbate underlying mental illnesses and/or behavioral disorders (MBDs), contributing to higher rates of mortality and morbidity among individuals with these conditions. Current diagnostic standards for mental illnesses use multiaxial criteria to assess individuals within the context of their condition among other contributing factors [9] which can allow physicians to identify environmental conditions that may exacerbate MBDs.

Studies have indicated that there is a relationship between high ambient temperatures and heat-related morbidity and mortality [10,11,12]. As the climate continues to warm, there may be more intense and frequent extreme heat events in the United States which may, in turn, impact the frequency of heat-related mortality and morbidity. A number of studies, highlighted in the National Climate and Health assessment, indicate that there is an elevated risk of mortality among people with MBDs associated with high ambient temperatures [13]. For example, a meta-analysis involving six case-control studies with 1,065 total patients indicated that among preexisting medical conditions, psychiatric illnesses were most strongly associated with heat-related mortality [14]. Additionally, a study in England showed a 3.1% increase in mortality per 1°C increase in heat for patients with mental illnesses [15]. A study in Catalonia, Spain of cause-specific mortality during heat waves showed a risk ratio of 1.30 (95% CI, 1.21–1.40) for heat-related mortality among individuals with mental and nervous system disorders [16]. Both of these studies had limited discussion as to the hypothesized reasons for the increased mortality among patients with MBDs, which may include schizophrenia, substance abuse, dementia, and other psychoses that may be subject to the impact of extreme weather events.

In addition to mortality, mental illnesses are important to consider among risk factors for heat-related morbidity with increased hospital admissions during extreme heat events [17]. There have been numerous studies from Australia, Canada, Israel and other countries examining the association between high ambient temperatures and hospitalizations related to MBDs indicating an increased risk of temperature related-deaths and hospitalizations, especially among those with dementia and schizophrenia [18–21]. Additionally, there is mounting evidence that certain medications used to treat MBDs can exacerbate effects on individuals using psychotropic and cardiovascular medications when exposed to extreme heat [22,23]. In contrast, there have been fewer studies from the United States examining these associations and those that exist have been limited to specific heat events or smaller case studies [24–26]. Not all studies examining temperature-morbidity relationships in the U.S. have found that individuals with an MBD are at an increased risk for hospitalization due to exposure to high temperatures [27]. This may require further examination of specific regional and population factors which could be sensitive to varying geographical, societal and cultural contexts [8]. Further characterization of socioeconomic, environmental, temporal and specific MDB diagnostic factors will be needed to assess specific vulnerabilities among affected sub-populations.

The aim of this study is to characterize the risk factors associated with hospitalizations among individuals diagnosed with a mental illness and/or behavioral disorder and a concurrent heat-related illness (HRI) compared to those hospitalized with just a mental illness and/or behavioral disorder, using data from the 2001–2010 US Nationwide Inpatient Sample (NIS). The U.S. Agency for Healthcare Research and Quality has estimated that mental health conditions are one of the five costliest conditions in the United States, with expenditures of $57.7 billion in 2006. Additionally, access to mental health services among rural populations is limited and the uninsured, who suffer a greater burden of mental health disparities, have increased barriers to care, including costs, and inability to receive medical care or prescriptions [28]. Given the burden of mental illness on healthcare within the United States, understanding the relationship between heat-related illnesses and MBDs will be important for public health officials and healthcare practitioners involved in providing supportive mental health treatment.

## Methods

### Data sources

The Healthcare Cost and Utilization Project (HCUP) NIS, 2001–2010, which was developed by the Agency for Healthcare Research and Quality (AHRQ), was used as the primary data source for this research. The NIS consists of data from approximately 8 million hospitalizations per year, about 20% of all hospital discharges in the United States [29]. The NIS includes information on patients’ demographic characteristics, hospital characteristics, primary and secondary diagnoses and procedures (coded according to the International Classification of Diseases, Ninth Revision (ICD-9-CM), the type and source of admission, discharge disposition, insurance status, total hospital charges, and length of stay. Patient income is recorded as median household income for patients’ zip-code by quartile and insurance status is described at Medicare/Medicaid, private insurance or HMOs, self-pay/uninsured or other. Other types of insurance include workers’ compensation, Title V (maternal and child health services), and other government programs. Variables identified for analysis follow research by Schmeltz (2015) which examined risk factors influencing heat-related illness hospitalizations within the NIS dataset [30]. Additionally, prior studies have identified race/ethnicity, income, age, gender, and insurance type as important factors influencing heat-related illness hospitalizations [31,32]. Patient comorbidities were coded and assessed using the AHRQ algorithms based on methods developed by Elixhauser, et al [33]. Stratification and weighting variables allow calculation of national estimates and account for the complex sampling design.

### Subjects and definitions

During the study period, comparisons for our analyses were between patients that were hospitalized for a MBD and a concurrent HRI compared to those patients who were hospitalized with only a MBD and without a concurrent HRI. The study population consisted of inpatients with a primary or secondary diagnosis (primary reasons for hospitalization) of a mental illness and/or behavioral disorder (ICD-9 codes 290–319), with any concurrent diagnosis of a heat-related illness, which are a spectrum of illnesses ranging from mild conditions such as heat cramps to more serious conditions such as heat stroke (ICD-9 codes 992.0–992.9 or E-Code 900.0). Concurrent diagnoses are those HRI diagnoses that are present at the same time as the MBD diagnosis and are recorded on the same diagnostic record, albeit in a different diagnostic position (e.g. tertiary). The categorization of mental illness and/or behavioral disorders (MBD) was chosen to be consistent with previous heat-related hospitalization studies examining mental and behavioral illnesses in relation to exposures to high ambient temperatures [18,34]. The categorization includes psychoses (ICD-9 codes 290–299), neurotic and personality disorders (ICD-9 codes 300–302), psychoactive substance abuse (ICD-9 codes 303–305), mental illness and behavioral disorders—adult onset (ICD-9 codes 306–311), mental illness and behavioral disorders—childhood onset (ICD-9 codes 312–316), and mental retardation (ICD-9 codes 317–319). Since approximately 95% of all HRI hospitalizations within the NIS dataset occurred during the summer months (May-September), all hospitalizations not occurring during these months were excluded from analysis. Additionally, of the cases analyzed, the majority (approximately 95%) were admitted through the emergency department with the remaining cases (approximately 5%) being admitted from another hospital or health facility.

### Statistical analysis

An initial descriptive analysis was performed on baseline inpatient sociodemographic and hospital characteristics for hospitalizations for HRIs and mental illness and/or behavioral disorders. Frequency counts of MBD hospitalizations with concurrent HRI were completed to assess distribution of specific MBD diagnoses within the study population. Separately, a log-binomial regression in a generalized linear mixed model (GLMM) and modified Poisson regression in a generalized linear model (GLM) were applied using a log link function and accounted for clustering. The regression coefficients from the log-binomial and modified Poisson regressions directly model relative risk (RRs) [35,36] and were used to calculate adjusted RRs for hospitalizations of MBDs with an HRI compared to hospitalizations of MBDs alone. The log-binomial regression was used with the full model for all MBDs while the modified Poisson regression was used to model the subsets of psychoses and psychoactive substance abuse diagnostic categories.

$$\text{log}\left(\pi_{i}\right)=\beta_{0}+\beta_{1}X_{1i}+\beta_{2}X_{2i}+\ldots +\beta_{K}X_{Ki}$$

Where (π_i_) is the probability of experiencing the outcome of interest, having a MBD with an HRI. β_1_X_1*i*_ …β_K_X_K*i*_ are the predictor variables, including gender, age, race, zip code income quartile, insurance status, hospital location, size and region, comorbid conditions, and year. The modified Poisson regression has the same form of the log-binomial equation, but uses a Poisson distribution instead of binomial [37]. Different models were employed to overcome convergence problems from the log-binomial distribution in the breakout of the psychosis and psychoactive substance models. Reference groups in all analyses were all hospitalizations for mental illnesses for which there was not an HRI diagnosis. All statistical analyses were performed using SAS 9.4 (SAS Institute, Cary, NC). This study conforms to the HCUP data use agreement.

## Results

There were a total of 37,019,792 hospitalizations during the study period, 2,872,636 were for mental illness and/or behavioral disorders and 14,948 cases were for HRIs. Of the primary or secondary mental illness and/or behavioral disorder diagnoses cases, 770 of those had a diagnosis of a heat-related illness, which was our main study population. This was approximately 0.03% of all MBD hospitalizations (and 5.2% of all HRI hospitalizations) during the study period. Patient characteristics of MBD hospitalizations with an HRI followed similar patterns to patients hospitalized with HRIs alone, including greater mean age, more likely to be male, residing in lower zip-code income quartiles (households at or below the 50th percentile for median household income in the U.S.), and being uninsured (Table 1).

**Table 1:** Patient and hospital characteristics for hospitalizations of heat-related illness and mental illness/behavioral disorders*.

	All Hospitalizations	Heat-Related Illnesses (HRI)	Mental Illnesses	Mental Illness and HRI
**N (Total, unweighted)**	37,019,792	14,948	2,872,636	770
**N (Total, weighted)**	181,094,795	73,180	14,148,649	3,782
**Age, Mean (±SD)**	47.7 (27.9)	55.0 (21.6)	45.0 (18.9)	51.3 (17.6)
**Age Categories, %**				
**0–17**	16.6%	4.4%	6.0%	1.8%
**18–39**	21.6%	20.5%	34.2%	23.1%
**40–64**	27.6%	38.2%	44.1%	52.4%
**65–74**	13.0%	13.4%	6.5%	9.8%
**75+**	21.3%	23.4%	9.2%	12.9%
**Gender, %**				
**Male**	41.3%	73.6%	51.6%	75.1%
**Female**	58.7%	26.4%	48.4%	24.9%
**Missing**	0.3%	0.1%	0.4%	-
**Race/Ethnicity, %**				
**White**	52.3%	55.4%	52.9%	52.6%
**African American**	11.2%	14.0%	13.9%	15.5%
**Hispanic**	10.4%	9.4%	7.6%	8.1%
**Asian/Pacific Islander**	1.8%	0.1%	0.8%	0.8%
**Native American**	0.4%	0.6%	0.4%	1.0%
**Other**	2.6%	2.2%	2.4%	2.4%
**Missing**	21.3%	18.2%	22.0%	19.7%
**Zip-Code Income Quartile, %**				
**0 to 25**^**th**^ **percentile**	22.9%	30.0%	25.8%	32.9%
**26**^**th**^ **to 50**^**th**^ **percentile**	20.9%	21.5%	20.7%	19.7%
**51**^**st**^ **to 75**^**th**^ **percentile**	18.7%	15.6%	17.6%	16.7%
**76**^**th**^ **to 100**^**th**^ **percentile**	16.1%	11.4%	13.8%	8.8%
**Missing**	21.4%	21.5%	22.1%	21.8%
**Payer—Primary, %**				
**Medicare/Medicaid**	56.0%	48.7%	53.6%	52.0%
**Private/HMO**	34.8%	27.8%	27.5%	19.3%
**Uninsured**	5.8%	15.0%	13.5%	22.7%
**Other**	3.2%	8.1%	5.1%	5.6%
**Missing**	0.2%	0.3%	0.3%	0.4%
**Hospital Region**^a^, **%**				
**Northeast**	17.1%	11.6%	15.30%	11.3%
**Mid-West**	20.0%	18.8%	23.50%	21.4%
**South**	45.6%	54.4%	45.20%	49.7%
**West**	17.3%	15.2%	16.20%	17.6%
**Hospital Size (by # of beds), %**				
**Small**	11.9%	17.1%	10.5%	13.1%
**Medium**	24.5%	26.6%	25.4%	29.0%
**Large**	63.3%	55.8%	63.8%	57.3%
**Missing**	0.3%	0.5%	0.3%	0.5%
**Hospital Location, %**				
**Rural Area**	12.1%	23.8%	11.0%	21.6%
**Urban Area**	87.6%	75.8%	88.7%	77.8%
**Missing**	0.3%	0.5%	0.3%	0.5%

^a^ Hospital regions defined by HCUP: https://www.hcup-us.ahrq.gov/db/vars/nis_stratum/nisnote.jsp

* Percentages may not add up to exactly 100% due to rounding.

The distribution of specific MBD diagnoses with concurrent heat-related illness is shown in Table 2. The majority, or 93.4%, of MBD hospitalizations with a concurrent HRI diagnosis fell into two categories: psychoses (i.e. dementia and schizophrenia), which comprised 45.1% of the hospitalizations, and psychoactive substance abuse (including dependent and nondependent alcohol/drug abuse), which accounted for 48.3%. Nondependent abuse of alcohol/drugs (ICD-9 codes 305.xx), however, accounted for over a third (35.5%) of all MBD hospitalizations with an HRI. Risk of hospitalizations for cause-specific MBDs with a concurrent HRI are also shown in Table 2. Similar to the frequency distribution of the diagnostic categories, regression analyses of hospitalizations for MBD with concurrent HRIs showed increased risks among dementia patients (RR = 1.84, 95% CI: 1.66, 2.03) and those with psychoactive substance abuse (RR = 1.38, 95% CI: 1.30, 1.47), which includes nondependent abuse of drugs/alcohol (RR = 1.84, 95% CI: 1.73, 1.98). While there was a 5% increased risk for hospitalization among schizophrenic disorder patients with an HRI, this estimate was not statistically significant (RR = 1.05, 95% CI: 0.95, 1.15).

**Table 2:** Counts and risk ratios (RRs) of hospitalizations due to a primary or secondary diagnosis of MBD with a concurrent HRI diagnosis, United States, 2001–2010 (Summer).

ICD-9 Code	Diagnostic Description	Count	Percent	RR^b^	95% CI
**290–319**	**Mental illness and behavioral disorders**	**770**	**100%**	**0.64**	**0.62, 0.66**
**290–299**	**Psychosis**	**347**	**45.2%**	**0.54**	**0.51, 0.58**
290, 293–294	Dementias	89	25.6%	1.84	1.66, 2.03
291–292	Alcoholic/drug psychoses	84	24.3%	0.96	0.87, 1.07
295	Schizophrenic disorders	107	30.8%	1.05	0.95, 1.15
296	Episodic mood disorders	40	11.5%	0.21	0.19, 0.24
297–299	Other nonorganic psychoses	27	7.8%		
**300–302**	**Neurotic and personality Disorders**	**23**	**3.0%**	**0.34**	**0.28, 0.41**
**303–305**	**Psychoactive substance abuse**	**372**	**48.3%**	**1.38**	**1.30, 1.47**
303	Alcohol dependence	75	20.2%	0.67	0.61, 0.74
304	Drug dependence	24	6.5%		
305	Nondependent abuse of drugs	273	73.3%	1.84	1.73, 1.98
**306–311**	**Mental illness and behavioral disorders (adult)**	**19**	**2.5%**	**0.49**	**0.43, 0.57**
**312–316**^a^	**Mental illness and behavioral disorders (childhood)**	**<10**	**-**	**-**	**-**
**317–319**^a^	**Mental retardation**	**<10**	**-**	**-**	**-**

^a^ In accordance with HCUP data use agreement, no cells with counts under 10 are reported.

^b^ Risk ratios (relative risk) compared hospitalizations of MBDs with an HRI to hospitalizations of only an MBD during the study period.

Table 3 provides a summary of the risk factors associated with hospitalizations due to mental illness and/or behavioral disorders with a concurrent heat-related illness diagnosis. Risk factors by patient characteristics showed increased risk among African Americans (RR = 1.16, 95% CI: 1.11, 1.22) and Native Americans (RR = 1.70, 95% CI: 1.46, 1.99), compared to Whites. Males had elevated risks (RR = 3.06, 95% CI: 2.94, 3.20) compared to females, as did all adults 40 years and older (40–64 year olds: RR = 1.90, 95% CI: 1.81, 1.99; 65–74 year olds: RR = 2.94, 95% CI: 2.72, 3.16; and 75+ year old: RR = 2.58, 95% CI: 2.40, 2.78), compared to adults 18–39 years old. Additionally, patients hospitalized with a MBD and HRI from lower zip-code income quartiles (0-25th, 26th-50th, and 51st-75th percentiles) had increased risks compared to those in the highest zip-code income quartile (76th-100th percentile). Being uninsured was also a risk factor for hospitalizations among MDB patients with an HRI diagnosis during this time period, compared to patients with private health insurance (RR = 1.92, 95% CI: 1.82, 2.04). Hospital characteristics, including region, size and location determined additional risk associated with MBD and HRI hospitalizations. Patients in the Mid-West (RR = 1.97, 95% CI: 1.84, 2.12), South (RR = 2.19, 95% CI: 2.07, 2.32) and West (RR 1.51 95% CI: 2.98, 3.18) had higher risks compared to the Northeast region. Among patients with an MBD and HRI hospitalizations in rural areas also showed increased risks compared to those in urban areas (RR = 2.24, 95% CI: 2.14, 2.35).

**Table 3:** Multivariable model of risk factors for hospitalization for all mental illness and/or behavioral disorders, psychoses, and psychoactive substance abuse with a concurrent HRI.

Characteristics	Mental Illness and Behavioral Disorders		Psychoses		Psychoactive substance abuse	
	RR	(95%CI)	RR	(95%CI)	RR	(95%CI)
**Race**						
White	*ref*.		*ref*.		*ref*.	
African American	**1.16**	**1.11, 1.22**	0.99	0.98, 1.00	**1.09**	**1.08, 1.10**
Hispanic	0.96	0.90, 1.03	0.95	0.94, 0.96	0.99	0.98, 1.00
Asian/Pacific Islander	0.98	0.82, 1.17	**1.07**	**1.06, 1.09**	0.76	0.75, 0.78
Native American	**1.70**	**1.46, 1.99**	0.93	0.91, 0.96	**1.17**	**1.14, 1.19**
Other	0.99	0.88, 1.11	0.98	0.97, 0.99	0.97	0.96, 0.98
**Gender**						
Female	*ref*.		*ref*.		*ref*.	
Male	**3.06**	**2.94, 3.20**	0.97	0.96, 0.98	**1.41**	**1.40, 1.42**
**Age Group (years)**						
0–17	0.71	0.63, 0.82	0.90	0.89, 0.91	0.30	0.29, 0.31
18–39	*ref*.		*ref*.		*ref*.	
40–64	**1.90**	**1.81, 1.99**	0.98	0.97, 0.99	0.99	0.98, 0.99
65–74	**2.94**	**2.72, 3.16**	0.95	0.94, 0.96	0.72	0.71, 0.73
75+	**2.58**	**2.40, 2.78**	**1.18**	**1.17, 1.19**	0.23	0.22, 0.24
**Zip-Code Income Quartile**						
0-25^th^ percentile	**1.42**	**1.33, 1.51**	0.96	0.96, 0.97	**1.10**	**1.09, 1.11**
26^th^ to 50^th^ percentile	**1.23**	**1.15, 1.31**	0.95	0.94, 0.96	**1.07**	**1.06, 1.08**
51^st^ to 75^th^ percentile	**1.30**	**1.22, 1.39**	0.97	0.96, 0.97	**1.04**	**1.03, 1.05**
76^th^ to 100^th^ percentile	*ref*.		*ref*.		*ref*.	
**Insurance Status**						
Medicare/Medicaid	1.02	0.98, 1.08	**1.30**	**1.29, 1.31**	0.83	0.82, 0.83
Private/HMO	*ref*.		*ref*.		*ref*.	
Uninsured/Self-pay	**1.92**	**1.82, 2.04**	0.90	0.89, 0.90	**1.26**	**1.25, 1.27**
Other	1.06	0.97, 1.16	1.01	1.00, 1.02	**1.09**	**1.08, 1.10**
**Hospital Region**						
Northeast	*ref*		*ref*.		*ref*.	
Mid-West	**1.97**	**1.84, 2.12**	**1.06**	**1.05, 1.06**	0.91	0.90, 0.92
South	**2.19**	**2.07, 2.32**	**1.02**	**1.01, 1.02**	0.89	0.88, 0.89
West	**2.98**	**2.78, 3.18**	0.92	0.91, 0.92	0.98	0.97, 0.98
**Hospital Size**						
Small	**1.47**	**1.39, 1.56**	0.95	0.95, 0.96	**1.06**	**1.05, 1.07**
Medium	**1.28**	**1.23, 1.33**	1.00	0.99, 1.01	**1.03**	**1.02, 1.04**
Large	*ref*.		*ref*.		*ref*.	
**Hospital Location**						
Rural Area	**2.24**	**2.14, 2.35**	0.92	0.91, 0.93	**1.01**	**1.00, 1.02**
Urban Area	*ref*.		*ref*.		*ref*.	

Specific diagnoses of psychoses (ICD-9 codes 290–299) with a heat-related illness showed that Asian/Pacific Islanders (RR = 1.07, 95% CI: 1.06, 1.08) had increased risk for hospitalization compared to Whites. Adults 75 years and older (RR = 1.18, 95% CI: 1.17, 1.19) had an elevated risk of hospitalization, compared to 18–39 year olds for a psychoses diagnosis and concurrent HRI. Higher risk for MDB and HRI hospitalizations were also seen among diagnosed psychoses patients with Medicare/Medicaid coverage, compared to patients with private health insurance (RR = 1.30, 95% CI: 1.29, 1.31) and among patients in the Mid-West and South, compared to the Northeast (Table 3)

Hospitalizations for psychoactive substance abuse with heat-related illness showed elevated risks among a few groups, including African Americans (RR = 1.09, 95% CI: 1.08, 1.10) and Native Americans (RR = 1.17, 95% CI: 1.14, 1.19), compared to Whites. The frequency of observations was very small within racial groups for psychoactive substance abuse and psychoses, even when weighted. This was especially true for the Asian/Pacific Islander (n<15) and Native American (n<15) groups. Males (RR = 1.41, 95% CI: 1.40, 1.42), compared to females, the uninsured (RR = 1.26 95% CI: 1.25, 1.27), and patients with ‘other’ types of insurance (RR 1.09, 95% CI 1.08, 1.10), compared to patients with private insurance, all had increased risk of hospitalization among psychoactive substance abuse patients with a concurrent heat-related illness. Similar to hospitalizations for all MBDs with an HRI, greater risks for hospitalization were seen in patients from lower zip-code income quartiles among patients diagnosed with psychoactive substance abuse and a concurrent HRI diagnosis, compared to those in the highest zip-code income quartiles. Hospital characteristics such as location and number of inpatient beds were also risk factors for hospitalizations of patients with psychoactive substance abuse with an HRI diagnosis compared to their referent groups (Table 3).

## Discussion

Our results suggest that several mental illness and/or behavioral disorder hospitalizations are associated with concurrent diagnoses of hospitalizations for a heat-related illness. There is a significant association among psychoses and psychoactive substance abuse diagnoses with concurrent heat-related illness diagnoses, particularly dementias and nondependent abuse of drugs/alcohol, compared to those hospitalized and diagnosed with psychosis or psychoactive substance abuse alone. Other published literature, on temperature-related mortality and hospital admissions from Canada, Vietnam, Australia, and the United Kingdom have also identified specific MBD’s associated with exposure to high ambient temperatures [18,19,38,39]. However, to our knowledge, this study is the first to use a nationally representative sample of hospitalizations from the United States to identify risk characteristics associated with hospitalizations for MBDs and concurrent HRI diagnoses compared to just MBD hospitalizations, although without a temperature-related exposure. Risk factors for hospitalizations of MBDs with a concurrent HRI followed similar patterns to prior studies examining heat-related illness hospitalizations, though there were notable differences [31,40].

While African Americans are a known vulnerable group for HRI hospitalizations, in our study Native Americans are also at a greater risk for hospitalization due to MBDs and a concurrent HRI diagnosis compared to all other MBD hospitalizations. Native Americans share a disproportionate burden for mental illnesses in the Unites States [41], and studies have also shown they are more likely than most other racial/ethnic groups to have substance-related disorders and to be hospitalized for alcohol and drug use disorders in the U.S. [42,43]. This finding suggests that specific interventions that target Native American mental health services could also incorporate educational information on the hazards that exposure to high ambient temperatures may have on this population. Increasing rural mental health services, specifically among Native American populations, could also help to improve knowledge and treatment to reduce these disparities. Our results for psychoactive substance abuse hospitalizations with concurrent HRI also indicate that males, compared to females, had a higher risk for hospitalizations among MBD patients. By contrast, a study by Hansen, et al., suggested that females diagnosed with psychoactive substance abuse had an increased risk of death during heat waves [18]. There may be significant gender differences among individuals who are hospitalized versus individuals who die during extreme heat events, specifically for those with psychoactive substance abuse diagnoses. These differences could be related to specific behavioral or occupational factors, as males are more likely to have substance abuse issues in adulthood [44,45] as well as being more likely to work in outdoor environments, increasing their heat exposure. Other studies have also noted specific gender difference in heat-related morbidity and mortality and similarly conclude differences in occupation, recreational activities, or healthcare utilization may be causal factors, though noncausal factors are also possible [46,47]. These differences need to be explored further to determine specific gender relationships among heat-related MBD hospitalizations. Additionally, our results also indicate that some diagnoses of MBD show a ‘protective’ effect, with the risk of hospitalization lower among MBDs with a concurrent HRI compared to hospitalizations of an MBD alone (for example, episodic mood disorders and other nonorganic psychoses). This could be due to the small sample size among these diagnoses. Although high ambient temperatures can exacerbate conditions, requiring hospitalizations, we do not see an increase in risk for hospitalizations among some of the MBDs; these include episodic mood disorders, neurotic and personality disorders, other non-psychotic disorders, and intellectual disabilities. It may also be that some MBDs are not impacted by extreme heat and further investigations into specific diagnoses of MBDs and high ambient temperatures are needed to examine these relationships.

Additionally, individuals hospitalized for psychoactive substance abuse may have impaired physiological response to high ambient temperatures. Opiates and alcohol can interfere with the body’s ability to thermoregulate, increasing cutaneous vasodilation and perspiration, causing dehydration [19,39,48]. Kidney function is also impacted from substance abuse and there is an association between renal dysfunction and extreme heat [49,50]. Individuals who are hospitalized for psychoactive substance abuse may also be at an increased risk for renal disease, or may be vulnerable to a heat-related illness hospitalization due to comorbid renal disease. While substance abuse has been associated with high ambient temperatures in only a few studies, these risk factors may contribute to overall hospitalizations in this population. Our results lend evidence to and agree with findings from similar studies examining the relationship between high ambient temperatures and substance abuse and there may be an increased need for public health interventions which specifically address MBDs and psychoactive substance abuse during heat events in addition to the impacts of kidney dysfunction [19,50,51]. There may also be cognitive impairments, due to substance abuse, which can reduce an individual’s awareness of their surroundings and reduce their ability to perform adaptive behaviors such as drinking water, removing themselves from an exposed environment and accessing a cooler, air conditioned space [18,24]. While not explicitly examined in this study, comorbid conditions, such as cardiovascular diseases, diabetes, and respiratory diseases may have a modifying effect on the hospitalization of individuals with MBDs and concurrent HRIs as the aforementioned comorbid conditions are also known to be risk factors for heat-related illness hospitalizations [32].

Our results suggest an increased risk of hospitalization among diagnoses of psychoses with concurrent HRIs, including dementia and schizophrenia, which may have similar symptoms of cognitive impairment as those with psychoactive substance abuse. Our findings show no statistically significant increase in schizophrenia hospitalizations with an HRI. One study on temperature-mortality indicated that death rates increase among those with schizophrenia when temperatures increase [39], though Hansen et al., have noted the highly speculative nature of varying morbidity and mortality patterns among this population, which may require further investigation [18]. Our examination of hospitalizations for psychoses with concurrent HRI also indicates that the elderly and those using Medicare/Medicaid are at an increased risk for hospitalizations. This could be related to dementia diagnoses among the elderly and those on Medicare, as Alzheimer disease dementia is prevalent in this population with estimates of 4.1 million individuals aged 75 years or older, in 2010, suffering from this disease and projections of up to 7.0 million Alzheimer disease dementia cases in individuals aged 85 years or older by 2050 [52]. As the U.S. population continues to age, increased cases of dementia and the projected increases in ambient temperatures may lead to a greater number of hospitalizations among the elderly for MBDs with a concurrent heat-related illness.

A contributing factor for MBD hospitalizations with an HRI may be due to medications that are used to treat a variety of mental illnesses and other chronic conditions. Psychotropic drugs and a number of medications for cardiac conditions alter the body’s ability to thermoregulate. These types of medications have been suggested as one of the causes for heat-related hospitalizations and mortality [22,53,54]. While we were not able to evaluate the use or the number of medications prescribed, the use of illicit and prescribed medications may account for MBD hospitalizations with concurrent HRI. Elderly patients on antipsychotics for dementia and others being treated for various mental health conditions may also be taking multiple medications to treat underlying health issues, which could also affect the thermoregulatory response [23]. While these effects may not be apparent during cooler seasons, healthcare practitioners may need to evaluate the use of certain medications among patients suffering from MBDs during summer months and monitor them closely during periods of excessive heat. This can include warnings on prescription instructions/bottles or from outreach within the pharmacy community by making patients with certain prescriptions aware of the risks of taking certain medications during extreme heat events.

The results obtained suggest that among MBD patients, those who were low-income and uninsured were more likely to have an HRI than those MBD patients who were not low-income and uninsured. This follows similar patterns from other studies examining heat-related illness hospitalizations [31,40]. Though patients hospitalized who are low-income or of low socioeconomic status may also have additional or undiagnosed medical conditions due to a lack of access to or lack of affordable healthcare and additional analyses of these factors are needed to accurately assess relationships between low-income, low socioeconomic individuals and heat-related illnesses. Hospital characteristics indicate that small and medium sized hospitals (determined by number of hospital beds and site of hospital in either rural or urban areas) in the South and Mid-West sections of the U.S. had MBD patients that were at an increased risk for MBD hospitalizations with concurrent HRI. This may be due to the increased risk seen among rural populations, though further research is needed to elucidate what specific factors may be contributing to hospitalizations in these areas. Contrary to our results, it has been suggested that populations in urban areas, particularly the homeless, may be at an increased risk of heat-related hospitalizations, especially due to the high incidence of mental illness and/or behavioral disorders within that population [55]. Additionally, factors like the urban heat island effect may impact this population more so than other urban populations due to their increased exposure to the environment and lack of access to protective factors in urban areas, such as air conditioning. While there have been efforts during the winter months to protect the homeless from extreme cold, these efforts could also apply to homeless populations during extreme heat events as well. This could be achieved by increasing the number of homeless shelters that remain open, identifying cooling centers, providing free public transportation, and increasing outreach programs since access to traditional mass media and information on the internet are not available to most homeless persons. Increased risk of hospitalization was seen in the Mid-West and South, with no significant risk for rural populations among patients with psychoses and concurrent HRI. Elevated risks were also seen among all MBDs with an HRI, compared to MBD hospitalizations alone, that show a slight increase in risk among those with psychoactive substance abuse and HRI among those who live in rural areas. This finding may suggest specific regional differences in heat-related MBD hospitalizations. While our data did not allow examination of other geographical and environmental variations in MBD hospitalizations and concurrent HRI with greater accuracy due to small sample size, further research is needed to examine the potential regional and local differences among populations with MBDs susceptible to heat-related hospitalizations. This finding could help public health services, especially those providing mental health and outreach services, to identify vulnerable populations and implement necessary plans to improve health disparities and reduce hospitalizations across different regional and urban/rural locations.

Given that this is one of the few studies examining hospitalizations for specific mental illnesses and behavioral disorders associated with concurrent heat-related illness in the U.S., there are a number of limitations to this study. Our dataset does not capture date of admission so we were unable to link a specific temperature exposure to the heat-related illness diagnoses. Since 95% of all HRI hospitalizations occurred during the summer months (May—September), we limited our examination to this time period as a proxy, though some heat-related illnesses may be unrelated to high temperature exposures. Our data also assume correct diagnostic coding for HRIs and MBDs. Administrative data are not as accurate as clinical data, and there may be additional errors in diagnostic coding and underreporting of heat-related illnesses. Psychiatric illnesses that are severe enough may take precedence over the coding of a heat-related illness, though as with any hospitalization, there may be unrecorded factors that influence why an individual was hospitalized and how diagnoses were coded. Additionally, the NIS data do not include information on possible confounders associated with heat-related illness hospitalizations such as air pollution, health status or activity prior to hospitalizations, or medications, which may be important effect modifiers for outcomes associated with heat-related morbidity. Populations, such as the homeless, which are at risk for heat-related illnesses due to their environmental exposures and health disparities, were not examined in the study due to the NIS not capturing patient living situation. And although this population was not examined in our analysis, they remain a high risk group and further information on homelessness and the impacts of extreme heat need to be further studied. We also acknowledge the limited sample size of our data, particularly the examination of specific patient characteristics among the analyses of psychosis and psychoactive substance abuse diagnoses. In addition to the limited sample size, some variables, including race/ethnicity and zip-code income quartile, have higher percentages of missing data (approximately 20%). We did not employ interpolation methods so we advise caution when interpreting the results from these analyses. Further research, including larger patient populations, medication use among those diagnosed with MBDs, and assessment of behaviors or activities prior to and during heat events will help to improve understanding of risk factors for heat-related illness hospitalizations among the mentally ill.

## Conclusions

Individuals with mental illnesses and behavioral disorders may be at a greater risk for hospitalizations with concurrent heat-related illnesses. Many people with MBDs could be susceptible due to socioeconomic status, race/ethnicity, health status and medication use, as well as the inability to recognize the risk of exposure to extreme heat. Preventative measures should be developed with this population in mind to reduce the threat of serious illness and minimize hospitalizations. Improved public health services can take the form of outreach to vulnerable subpopulations, such as the homeless and those with substance abuse issues; patient education during healthcare visits to assess medication use; or the integration of heat health information into social services among susceptible populations, including the elderly with dementia. Hospitalizations are expected to increase as high temperatures and extreme heat events continue to rise along with the frequency and intensity associated with the impacts of climate change. Our findings highlight the need for additional research in this area, but also identify certain risk characteristics among patients with MBDs and concurrent HRIs that can be used to prevent future hospitalizations for high ambient temperatures related to a changing climate.
